# Experimental Investigation of Delayed Fracture Initiation in Advanced High-Strength Steel Under Accelerated Bending

**DOI:** 10.3390/ma18143415

**Published:** 2025-07-21

**Authors:** Kyucheol Jeong, Jaewook Lee, Jonghun Yoon

**Affiliations:** 1 Department of Mechanical Engineering, Hanyang University ERICA, 55 Hanyangdaehak-ro, Sangnok-gu, Ansan-si 15588, Republic of Korea; 2 BK21 FOUR ERICA-ACE Center, Hanyang University, 55 Hanyangdaehak-ro, Sangnok-gu, Ansan-si 15588, Republic of Korea; 3 Materials Forming Research Group, POSCO Global R&D Center, 100 Songdogwahak-ro, Yeonsu-gu, Incheon 21985, Republic of Korea

**Keywords:** advanced high-strength steel (AHSS), bending failure, strain rate, rate-dependent fracture

## Abstract

Predicting bending fractures in advanced high-strength steel (AHSS) is challenging due to complex microstructural behaviors and strain rate dependencies, particularly in industrial forming processes. Current models and standards primarily focus on quasi-static tension or slow bending speeds, leaving a gap in understanding the accelerated failure of AHSS without necking. In this study, direct bending experiments were conducted on dual-phase, complex-phase, and martensitic AHSS grades under varying bending speeds and radii. Since the bending crack is irrelevant to the load drop, surface crack evolution was measured using three-dimensional surface profile analysis. The results showed that accelerated bending significantly delayed crack initiation across all tested materials, with small-radius bending showing reduced strain localization due to strain rate hardening. Larger-radius bending benefited primarily from increased fracture strain.

## 1. Introduction

Bending is common in sheet-metal forming. However, predicting bending fractures in advanced high-strength steel (AHSS) remains indistinct and challenging due to through-thickness gradients, the formation of shear bands, and the production of localized shear-induced microcracks at the bending tip [1,2,3,4]. These fractures are induced by the hardness differences between multiple phases [5,6,7], which are the characteristics of many AHSS. Unlike tension tests, where failure is typically preceded by necking or load drops, bending tests often exhibit sudden surface crack initiation without any precursors. This behavior makes them difficult to model using constitutive models calibrated from tension-based tests, given the differing three-dimensional stress states [1,8]. As a result, bendability is typically assessed using direct bending tests rather than commonly used tension-based tests.

Many bending test standards do not consider the bending rate, and tests are usually conducted at slow speeds. For example, the VDA-238-100 test [9], a widely preferred standard in the automotive industry, specifies a bending punch speed of 20 mm/min. Although this speed is slightly above quasi-static conditions, it is much slower than those encountered in realistic, commercial forming processes. In industrial forming applications, punch speeds in forming operations, such as simple bending, flanging, or stretching, generally reach much higher values, though they typically do not exceed 1 m/s [10]. Speeds greater than 50 m/s are associated with high-velocity formation of metals with explosives [11]. Other standards, such as ISO-7438 [12] and ASTM-E290 [13], specify bending punch rates of 60 mm/min or do not provide specific guidance, respectively. Hence, the relationship between laboratory test results and production line performance remains unclear.

Most studies of rate dependency focus on uniaxial tension tests or notched tension tests, in which necking occurs before fracture. These studies reveal that rate dependency varies according to the type of AHSS and the fracture measures. Sato et al. [14] found that a reduction in the cross-sectional area of failed specimens in uniaxial tension is independent of strain rate for all tested steel types, including dual-phase (DP) and martensitic (MS) steels. In contrast, Kim et al. [15] reported that, for transformation-induced plasticity (TRIP) steels, area reduction is influenced by strain rate, with total elongation increasing at higher strains. Gronostajski et al. [16] reported that total elongation in DP and TRIP steels has a nonlinear relationship with strain rate due to the interplay between thermal effects and strain hardening at low strain rates. Roth and Mohr [17] and Erice et al. [18] developed models for AHSS that incorporate both thermal softening and rate-dependent hardening using a Johnson–Cook framework [19], with fracture strain increasing with strain rate [18]. These studies primarily focused on rate effects in tension-based tests, for which necking is an important factor. In tension-based tests, strain-hardening delays necking and alters stress triaxiality. The resulting fracture displacement is significantly coupled with hardening behavior. Under these conditions, it is hard to discuss bendability because bending does not exhibit significant necking or flow instability.

Meanwhile, only a few studies have explored the effect of bending rates on AHSS. Kim et al. [20] and Lee et al. [21] examined the effects of temperature and strain rate on DP steel using finite element analysis (FEA). Lee et al. [21] found that higher strain rates can delay bending fracture, while the inclusion of thermal effects may cause only minor delays. Although their study demonstrated high predictive accuracy, it primarily relied on plasticity models derived from tensile-based tests and lacked experimental validation on how bending speed influences fracture behavior under pure bending conditions, as opposed to stretching-bending scenarios.

Beyond the strain rate effect, it is known that stepwise incremental bending can influence the fracture occurrence. A common practice in manufacturing is to employ sequential incremental bending, which is believed to reduce the likelihood of cracking during the bending of AHSS. The observed benefits of this approach involve several factors: redistribution of dislocations to non-critical regions after deformation [22], reductions in friction coefficients, and changes in the contact conditions that delay localized thinning [23]. Additionally, sequential bending has been shown experimentally to significantly reduce springback [24]. This introduces a conflict with the benefits of sequential bending in terms of total process time. Conventional rate-dependent hardening and fracture strain models suggest that rapid bending is advantageous, assuming the findings of tension tests can be extrapolated to the bending process. Conversely, incremental bending, which requires slower processing times, provides formability benefits.

One final distinction between tension-based tests and AHSS bending is the detection of fracture. In tight-radius bending, a load drop does not necessarily indicate cracking, or cracking does not result in a load drop, because shear cracks evolve gradually [4], and factors such as limited die stiffness and lift-off phenomena [25]. Therefore, this research assesses bendability by directly tracking the evolution of surface cracks rather than relying on load drop.

Accurate process design for AHSS bending requires modeling that reflects fracture behavior under realistic forming conditions. However, there is a lack of experimental data on AHSS fracture at high bending speeds. Since surface cracking in bending occurs without necking and follows a distinct failure mechanism, the existing literature, largely based on tensile tests, does not adequately capture this behavior. This study addresses that limitation by experimentally investigating crack initiation during high-speed bending, providing foundational insight into the rate-dependent fracture mechanisms under pure bending conditions.

The article is organized as follows. Section 2 describes the experimental bending tests and the procedures for measuring cracks, using surface geometry only rather than load drop. Section 3 presents the results of the bending tests and crack measurements, along with an analysis of the macroscopic behavior that contributes to increased bendability. Section 4 summarizes the results and their implications.

## 2. Methods for Bending and Crack Measurement

### 2.1. Bending Test at Different Speeds and Radii

The AHSS materials tested were DP, complex-phase (CP), and MS steels, provided by POSCO, Incheon, Republic of Korea. DP steel consists of a soft ferritic matrix with hard martensitic islands. CP steel primarily contains bainite and martensite, with ferrite. MS steel is composed of martensite, mainly with little bainite or ferrite [26]. Among the three, MS steel exhibits the lowest ductility, while DP steel shows the highest total elongation due to its lower yield-to-tensile strength ratio. All three AHSS contain a large hardness difference between the ferrite and martensite, a source of the localized growth of voids in bending and resulting shear crack in bending [2]. Vivek et al. and Kyucheol et al. showed that the crack develops and growth due to the interface decohesion for DP steel [4,27]. For a higher martensite fraction, the martensite cracking initiates at the edge of the martensite [28].

The CP and MS steels had a thickness of 1.2 mm (denoted as 1.2T), while the DP sheet was 1.6 mm thick (1.6T). Thicker sheets are used for DP steel because surface cracks were not observed in 1.2T specimens using the VDA-238-100 setup, indicating that near-hemming processes are feasible with this thickness, and bending fractures are not a concern for DP 1.2T. Square specimens (60 mm × 60 mm) were prepared using waterjet cutting.

**Figure 1:**
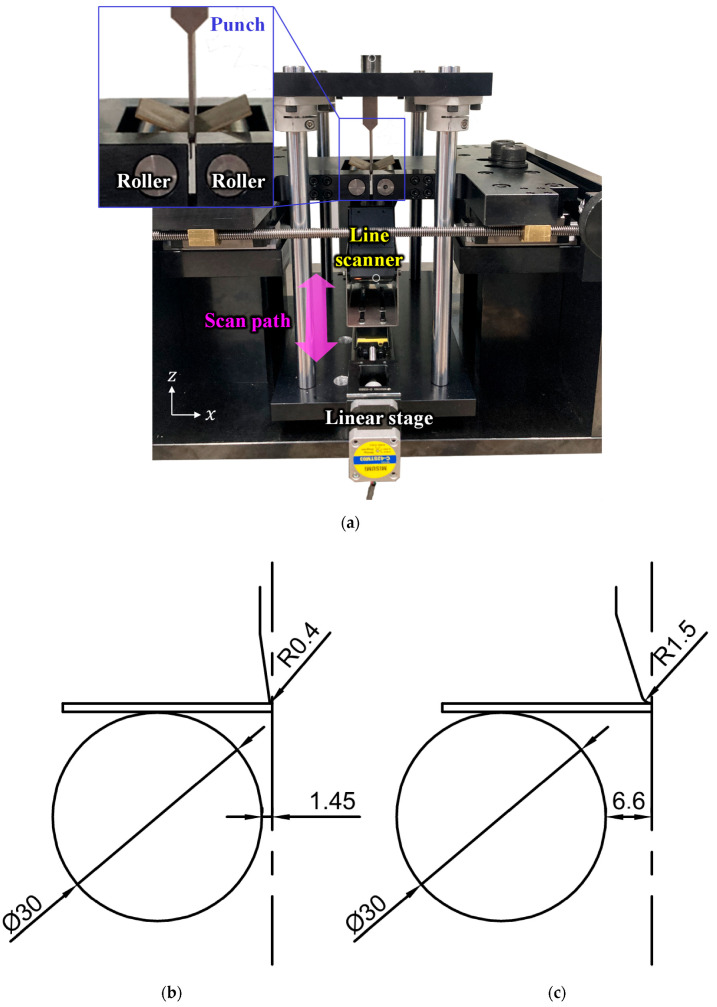
Experimental setup for three-point bending: (**a**) die setup for VDA-238-100; geometry of the punch and roller for (**b**) tight-radius and (**c**) large-radius tests (reprinted from [4] with permission from Elsevier).

Two bending setups with different punch radii were employed. The VDA-238-100 setup was used for tight-radius three-point bending (TR3PB). Figure 1a,b illustrate the die setup for the TR3PB test with 1.2T sheets. A larger-radius punch was also applied to the VDA-238-100 setup for large-radius three-point bending (LR3PB), as shown in Figure 1c. The DP steel was not tested in LR3PB due to its superior bendability. In the TR3PB setup, the distance between the rolls depended on the sheet thickness and was calculated as 0.5+2T in mm, where T represents the sheet thickness. In the LR3PB, the distance is 0.5+2T+10.3, with the additional 10.3 mm corresponding to the punch width. The line scanner was required for surface measurements (Section 2.2).

Two punch speeds were used in the tests. Slow-speed tests used 18 mm/min, closely matching the VDA-238-100 standard (20 mm/min). Accelerated tests used a punch speed of 480 mm/min. The speed of the bending punch was limited by the needle-like punch-die setup, as shown in Figure 1b,c, because further acceleration could lead to punch buckling or breakage. But the strain increment per stroke is relatively large in the TR3PB setup, as the R/T ratio ranges from 0.25 to 0.33, which is significantly lower than the typical industrial configurations where R/T is around 5. The strain rate sensitivity of AHSS in tensile tests is reported to be either monotonic [15] or nonlinear [16]. In this study, the nonlinear effects are not considered, and a monotonic trend is assumed as in [18].

Bending continued to the specific angle at which differences in crack evolution between the two speeds were observed. Each test was repeated five times for each material and speed to ensure consistent and reliable results.

### 2.2. Surface Measurements for Outer Radii and Cracks

For AHSS, observable bending cracks occur independently of the load drop in TR3PB [25] due to sudden shear cracking [3] and lift-off phenomena [29]. Consequently, previous studies have adopted manual identification of the crack with DIC images [8,30] or detection based on the onset of loss of correlation [31], both of which are sensitive to image resolution and crack visibility. In the present study, the onset of cracking was directly measured using a laser profile scanner to obtain surface profile data. The surface-scan inspection setup and method are described below and in detail by Jeong et al. [4]. No attempt was made to relate fracture to load drops in either TR3PB or LR3PB.

In our tests, the sheets were bent to the desired angle at different punch speeds, and the outer radius area of the specimen was scanned using a laser profiler scanner X8020 (Keyence, Osaka, Japan) with a resolution of 2.5 μm × 2.5 μm, generating three-dimensional (3D) surface profile data. In the raw scanned data, the sheet region was identified by its significantly higher luminance value compared to the dark-colored die (Figure 1a). The central 52 mm of the specimen, from the total length of 60 mm, was extracted for analysis. The width, or horizontal length in Figure 1b, of the region of interest was set as 4T/3. To isolate microcracks and surface waves with short wavelengths, a high-pass filter was applied to the scanned data, effectively removing macroscopic curvature caused by bending. The size of the Gaussian filter was set to 150 μm, which is several times larger than the expected crack width [4]. Regions where the depth values exceeded 1% of the sheet thickness were identified as microcracks. This threshold, proposed by Jeong et al. [4], balances noise reduction and detectability rather than assigning any specific physical significance to crack depth. Both the depth and area of these crack regions were extracted to evaluate crack evolution.

Using the surface-scanning method, the punch stroke corresponding to 1%T cracks was determined. Failure strain values and the corresponding punch strokes were taken from Jeong et al. [4]. These strain values were estimated based on FEA of the strain at the specified punch stroke. The time–average strain rate was obtained by dividing the strain by the time required for the bending (Table 1).

## 3. Results

**Figure 2:**
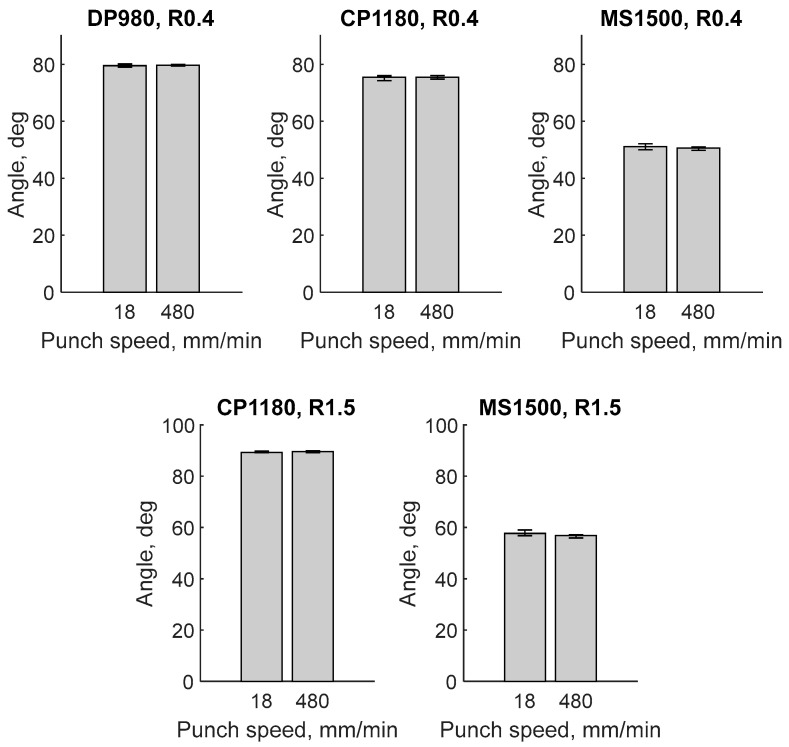
Validation of bending angle under tested conditions.

Because crack evolution varies with bending angle, it is important to validate the bending angle across punch speeds. Figure 2 and Table 2 depict the measured angle after bending. To obtain these measurements, a metal strip was stamped with paint, and the bending angle was determined using image analysis incorporating a Hough transform. The results confirm that the bending angles were identical for the two punch speeds, indicating that the punch stroke was controlled as intended, and any differences in springback between the two speeds could be ignored.

**Figure 3:**
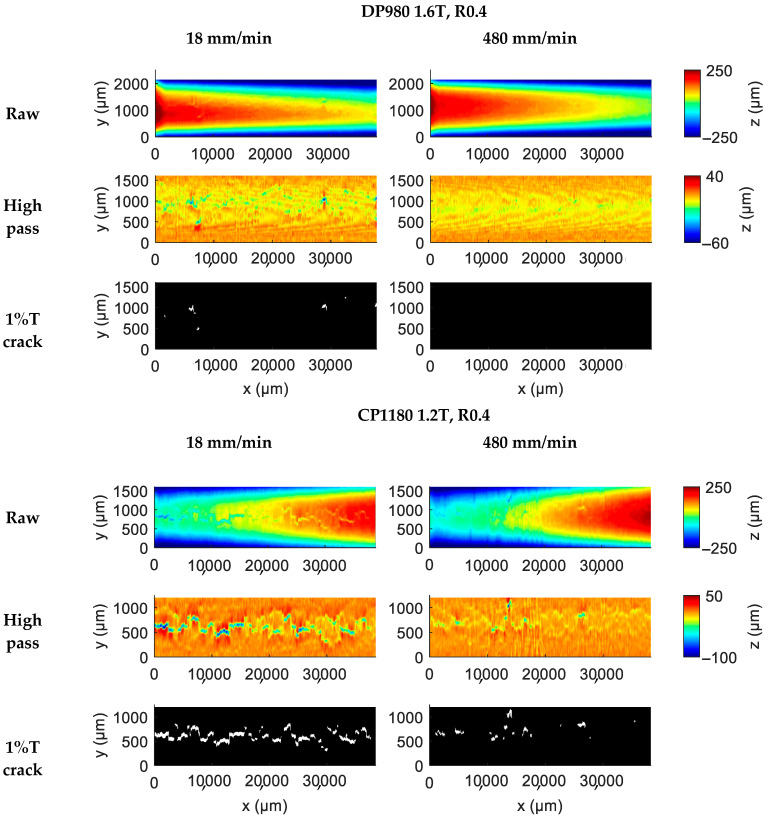

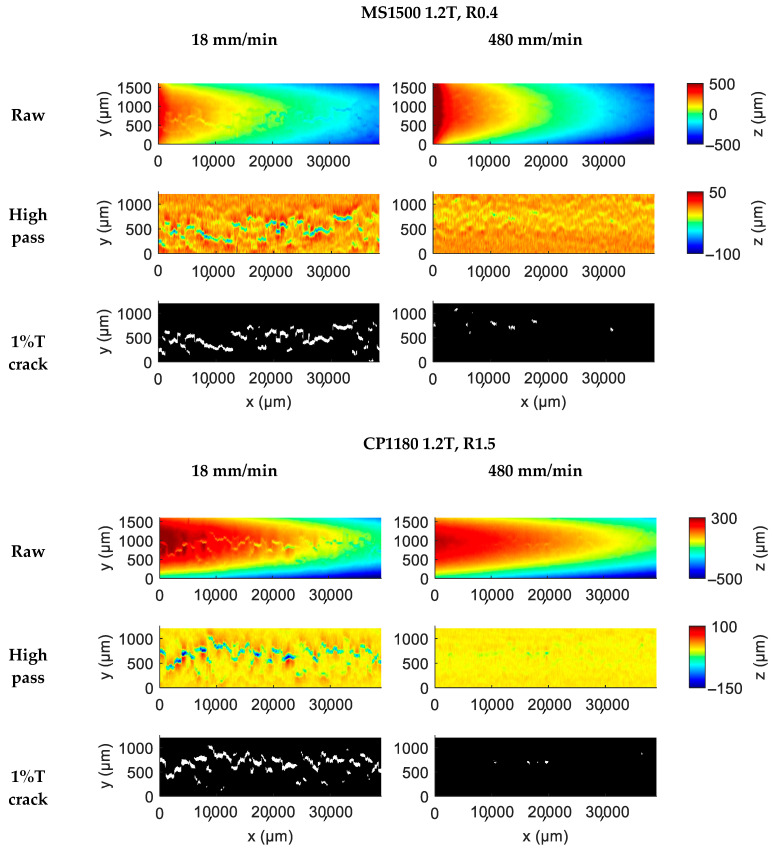

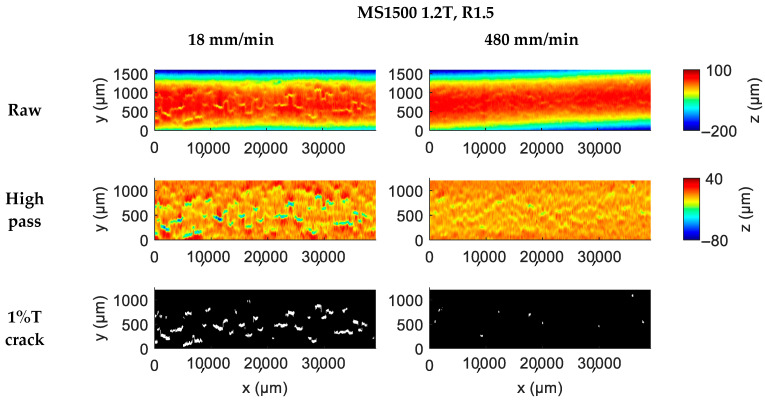
Result of surface profile analysis: raw, high-pass, 1%T filtered data for tested conditions.

Figure 3 illustrates the selected raw surface profile, high-pass-filtered surface profile, and the detected 1%T cracks at the bending angle shown in Figure 2. In the raw surface profile, the large-scale height gradient along the y-direction corresponds to the curvature of the bent sheet, while the gradient along the x-direction results from a height-leveling error in the linear stage. Both large-scale gradients are effectively removed by the high-pass filtering, leaving only small-scale surface defects. The 1%T crack map represents a thresholded version of the filtered data, where white pixels indicate regions exceeding the 1% thickness criterion, quantifying the severity and extent of surface cracking.

The results clearly show that crack evolution is significantly and consistently delayed at higher punch speeds, regardless of steel grade or punch radius. Each material exhibits distinct crack behavior. For example, DP980, despite having a smaller R/T ratio and a comparable bending angle to CP1180, shows much less crack development. At a punch speed of 480 mm/min, no bending cracks were detected in DP980. In contrast, MS steel demonstrates the highest susceptibility to bending cracks, due to its high martensite fraction and high yield-to-tensile strength ratio, both of which promote strain localization and early crack initiation.

**Figure 4:**
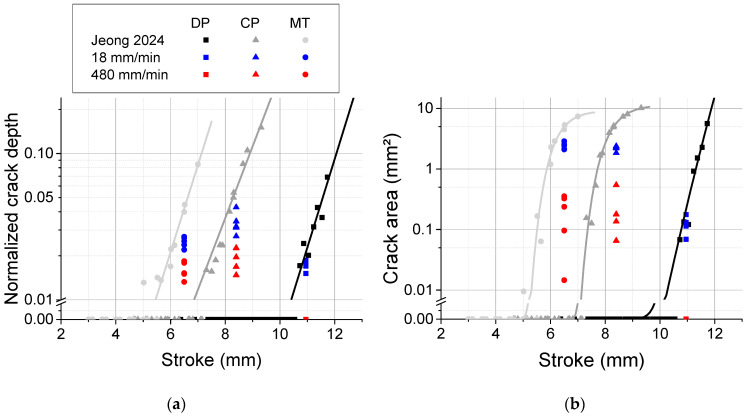

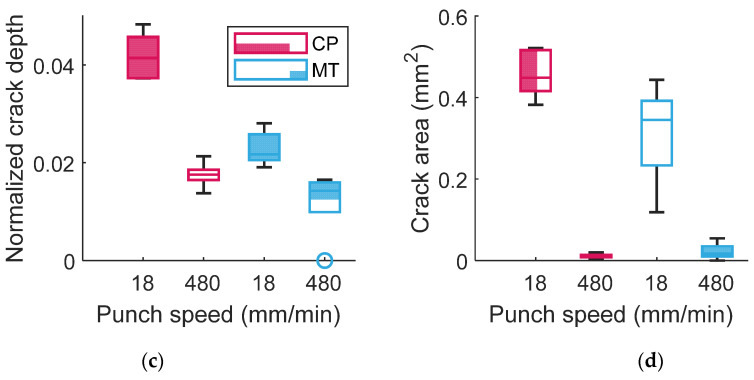
Evolution of cracks: (**a**,**b**) for TR3PB, including results from Jeong et al. [4], and (**c**,**d**) for LR3PB.

Figure 4 quantifies the evolution of crack depth with respect to the punch stroke. Open circles represent outliers with values below $Q_{1}-1.5\left(Q_{3}-Q_{1}\right)$, where $Q_{1}$ is the median of the lower half of the dataset and $Q_{3}$ is the median of the upper half. For TR3PB, the results from Jeong et al. [4] were adopted for comparison. Crack depth can be calculated from the root mean square of each pixel’s depth, normalized with respect to sheet thickness. The crack area corresponds to the area of the white pixels shown in Figure 3. The results clearly demonstrate that accelerated bending significantly reduced crack evolution.

Due to the complexity of the multiphase microstructures in various AHSS grades, the underlying microstructural mechanism contributing to enhanced bendability from accelerated punching is not yet fully understood. Nevertheless, our findings are consistent with previously proposed macroscopic models that describe the delayed onset of fracture at high strain rates [18]. This enhancement can be attributed to two primary macroscopic factors: altered material flow due to strain rate hardening and a direct increase in failure strain. A high strain-hardening exponent in bending results in a more uniformly formed radius and a delayed punch lift-off [25,29], both of which contribute to a larger radius. The stress increase with increasing strain rate and decreasing temperature is explained in terms of the energy required for dislocation obstacles [32,33].

**Figure 5:**
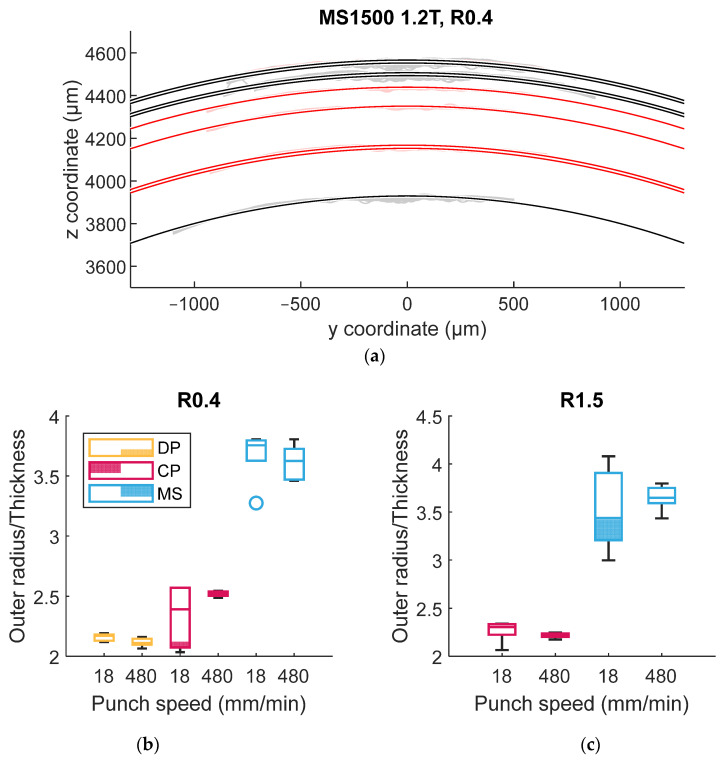
Measurement of outer radii with 3D profile data: (**a**) example of radii measurement; and outer radii of (**b**) TR3PB and (**c**) LR3PB.

To identify which mechanism primarily affects bendability, the outer radius of the bent specimen was measured, as this shape is influenced by strain hardening. The mean profile was extracted from the profile data in Figure 3 along the x-direction. Figure 5a provides an example of a calculation of the outer bending radius for MS steel with TR3PB. A single-curvature profile was obtained by applying a high-pass filter in the x-direction as in Figure 3, and the average profile was used for Pratt circle fitting [34]. Figure 5b,c depict the outer radii for all materials in TR3PB and LR3PB, respectively. For TR3PB (R=0.4 mm), an accelerated punch test resulted in a greater outer radius for CP steel (Figure 5b), indicating the effect of strain rate hardening. DP steel had a similar outer radius because the high bending angle approached a hemming-like shape. In this scenario, localized deformation effects diminish due to saturated strain hardening. In addition, DP steel reportedly exhibits minimum flattening during bending (i.e., local thinning at the tip) due to its relatively high strain-hardening capability [29]. For LR3PB (R=1.5), a slightly higher outer radius was observed with accelerated bending in MS steel, but the experimental scatter was larger than the observed differences, as shown in Figure 5c. Notably, the crack depth and area were significantly different and did not overlap (Figure 4c,d). This suggests that the primary mechanism enhancing bendability in all three materials was the increased failure strain ${\bar{\varepsilon }}_{f}^{p}\left(\sigma ,{\dot{\bar{\varepsilon }}}^{p}\right)$, with only a minor contribution from strain rate hardening, except in the case of CP steel in TR3PB.

**Figure 6:**
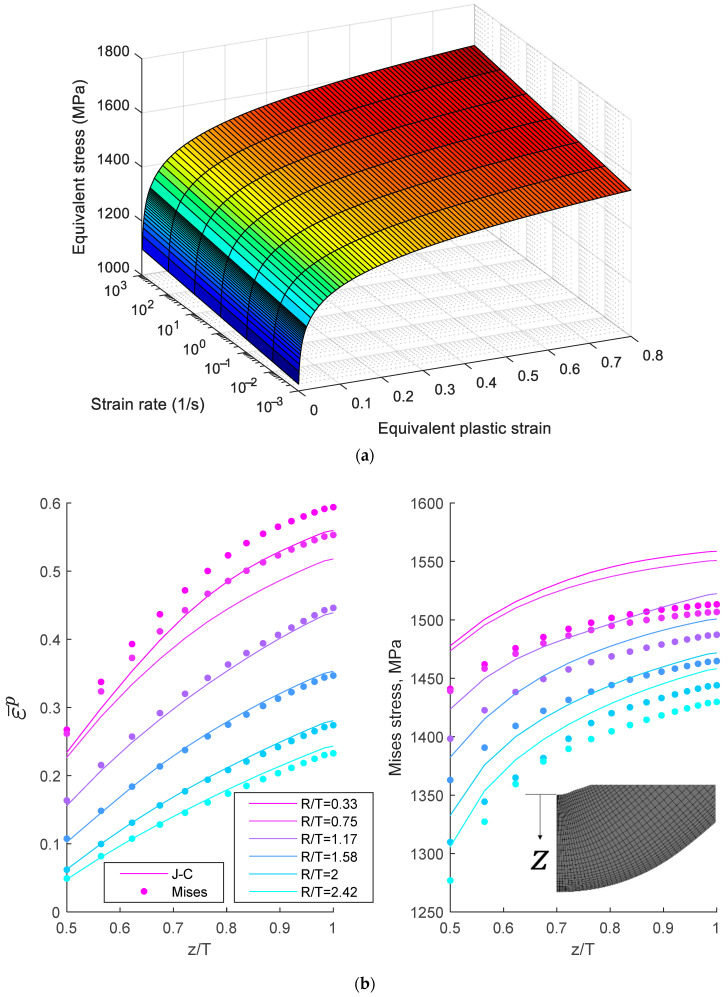
Effect of strain rate in a finite element analysis of high-speed three-point bending: (**a**) equivalent plastic strain (color scale represents stress) and (**b**) equivalent stress along thickness directions.

To further analyze the effect of strain hardening, strain values were compared using FEA for high-speed TR3PB. Figure 6a illustrates the strain rate dependency of CP1180 1.2T based on a Johnson–Cook model [19]. For simplicity, the yield condition was defined using the von Mises equivalent stress $\bar{\sigma }$ as

(1) $$f=\bar{\sigma }-\sigma_{r}\left({\bar{\varepsilon }}^{p},{\dot{\bar{\varepsilon }}}^{p}\right)=0$$

(2) $$\sigma_{r}=\left(a+b\left({\bar{\varepsilon }}^{n}\right)\right)\left(1+c\ln\frac{{\dot{\bar{\varepsilon }}}^{p}}{{\dot{\bar{\varepsilon }}}_{0}}\right)$$

The coefficients a=10, b=1551.77018 (in MPa), and n=0.06099 were obtained from uniaxial tensile tests conducted in accordance with the ASTM-E8 standard test [35]. The strain rate dependency of CP1180 1.2T, reported by Erice et al. [18], is represented by c=0.005049 and ${\dot{\bar{\varepsilon }}}_{0}=0.001$. Thermal softening was not considered, as the strain rates in this test were less than 1/s, whereas activation of thermal softening was calibrated at 3/s in the same reference. The TR3PB test, identical to that used in the experiment, was modeled using solid 3D elements with plane-strain boundary conditions. The punch radii and distances between rollers were 0.4+5N and 29+5N mm, respectively, where N=0, 1, 2, 3, 4, 5. The punch speed was fixed at 480 mm/s, and the bending process continued until a 90° bending angle was achieved. Figure 6b shows the strain and stress distributions along the thickness for the rate-dependent model (denoted as J-C) and the rate-independent model (denoted as Mises).

The results in Figure 6b indicate that, for VDA-238-100 tight-radius bending (R/T=0.33), the equivalent strain was lower when rate hardening was included. However, as the punch radius increased, the effect diminished because the strain distribution became nearly linear, transitioning to a pure bending state. For R/T≥1.17, the equivalent plastic strain was similar for the two models, although the difference in stress persisted. These findings align with the experimental results, which found that the effect of rate hardening was prominent only during tight-radius bending. This suggests that incorporating rate dependency into fracture strain is essential to accurately predict bending fractures, even at moderate speeds.

To summarize the results, bendability can be improved through punch control by applying a high bending rate. Therefore, determining the upper limit of punch speed at which these effects are sustained is essential for forming process design.

**Figure 7:**
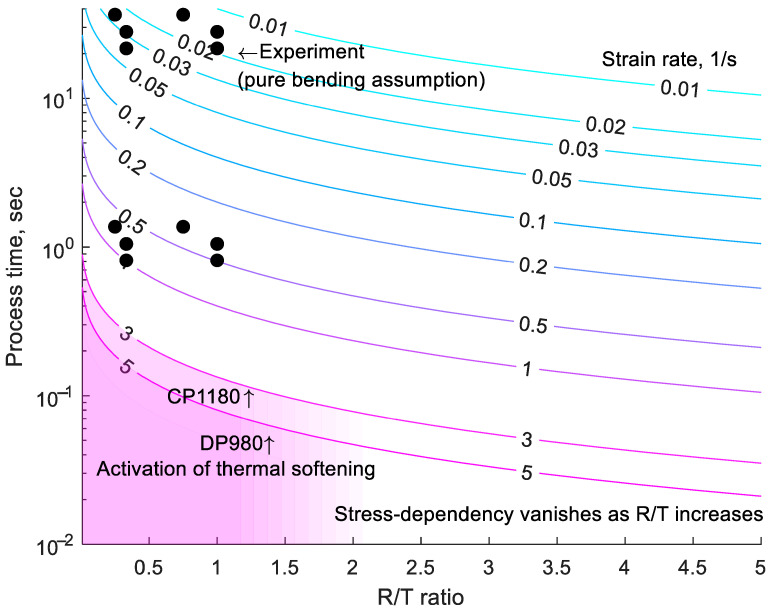
Example of a bending rate diagram for process design (color scale represents strain rate).

Strains in Table 1 were obtained using a hybrid approach combining experimental data and FEA, as introduced in Section 2. The strain rate can also be estimated using pure bending theory and plane-stress conditions, as described by Cai et al. [36] and Cheong et al. [37]. Assuming a simplified linear strain evolution, the strain rate can be calculated using the required process time and the following equations:

(3) $$\bar{\varepsilon }=\frac{2}{\sqrt{3}}\sqrt{\varepsilon_{I}^{2}+\varepsilon_{I}\varepsilon_{II}+\varepsilon_{II}^{2}\;}=\frac{2}{\sqrt{3}}\varepsilon_{I}≅\frac{2}{\sqrt{3}}\ln\frac{R_{o}}{R_{n}}≅\frac{1}{\sqrt{3}}\ln\frac{R_{o}}{R_{i}}≅\frac{1}{\sqrt{3}}\ln\left(1+\frac{T_{0}}{R_{i}}\right)$$

(4) $$\dot{\bar{\varepsilon }}≅\bar{\varepsilon }/\Delta t$$

where $\bar{\varepsilon }$ and $\dot{\bar{\varepsilon }}$ represent the effective strain and strain rate, respectively; $R_{o}$, $R_{n}$, and $R_{i}$ are the radii of curvature of the outer surface, neutral axis, and inner surfaces, respectively; and $T_{o}$ is the initial sheet thickness. Using Equations (3) and (4), the strain rate was estimated and plotted in Figure 7, with experimental points represented by dots. Note that the experimental strain rates of the experimental point were overestimated because fracture occurred during bending, and the inner bending radius did not achieve the punch radius. For example, MS steel, which had the lowest bendability among the tested AHSS, achieved $\bar{\varepsilon }=0.8$ in pure bending theory, but failed at approximately $\bar{\varepsilon }=0.33$.

The macroscopic mechanical effects of AHSS at a high strain rate include rate hardening, enhanced failure strain, and thermal effect. The strain rate in this study did not exceed 3–5/s, which is the threshold for thermal softening activation for CP1180 and DP980, as suggested by Erice et al. [18]. Therefore, the effect of thermal softening was not considered. However, if the strain rate exceeds this range, a thermal softening mechanism may be activated, negatively affecting bendability and counteracting the benefits of rate hardening in TR3PB. This stress-dependency is expected to diminish as the R/T ratio approaches pure bending. Beyond three-point bending tests, the limiting strain rate may also depend on geometry due to heat diffusion and inertia. Additionally, significant heat generation may influence phase transformation, as demonstrated in tensile tests by Gronostajski et al. [16].

## 4. Conclusions

This study experimentally validated the increased bendability of AHSS at different punch speeds. Using 3D profile analysis, the evolution of microcracks was examined, and the macroscopic mechanisms were discussed in relation to the rate hardening effect. The major conclusions are as follows:

- Accelerating the bending process delays the measured evolution of surface cracks. This trend was observed in DP, CP, and MS steels for both small-radius and large-radius bending.
- Unlike tension-based tests, bending tests allow the failure limit to be addressed without necking or flow instability. The enhanced bendability is attributed to an increase in failure strain rather than depending on the rate hardening (such as lift-off) since the bending radii were similar between the two speeds for DP and MS steel.
- For CP steel in tight-radius bending, accelerated bending produced larger bending radii, implying a significant effect of rate hardening. This indicates that accurate modeling of CP steel bending requires incorporating both rate hardening and rate-dependent fracture strain.
- Using pure bending theory, the process time limit beyond which bendability improvements are no longer guaranteed can be estimated. As discussed in Section 3, this limit is assumed to correspond to the strain rate at which thermal activation begins.
- Furthermore, our results suggest that the benefits of stepwise bending observed in industrial practice are due to relaxation or heat dissipation, rather than a direct strain rate effect itself. This distinction is important for interpreting the mechanism behind improved bendability in slow, incremental forming operations. However, additional experiments are needed to isolate and quantify the influence of relaxation effects.

## Figures and Tables

**Table 1:** Estimated strain rate for TR3PB.

$\mathrm{Strain}\;\mathrm{Rate}\;{\bar{\varepsilon }}_{f}^{p}/\Delta t$	DP980 1.6T	CP1180 1.2T	MS1500 1.2T
18 mm/min	0.0224	0.0104	0.0095
480 mm/min	0.5968	0.2773	0.2543

**Table 2:** Validation of bending angle under tested conditions (see Figure 2).

Punch radius (mm)	0.4						1.5			
Material	DP980		CP1180		MS1500		CP1180		MS1500	
Punch speed (mm/min)	18	480	18	480	18	480	18	480	18	480
Angle (degree)	79.5524	79.3609	75.8111	76.0552	52.0668	50.603	89.1062	89.9101	59.02	56.9368
	79.7246	79.8324	75.9962	75.5896	50.7595	51.0451	89.1015	89.2225	58.8665	57.0554
	79.04	79.359	75.9236	74.801	50.0062	50.7889	89.8222	89.8353	56.8551	57.2111
	79.0214	79.6706	75.1943	75.8007	50.8385	49.7906	89.1646	89.1777	56.9284	57.0547
	80.1123	79.9465	74.2204	74.9536	51.7876	50.8063	-	89.7726	56.8024	55.9274
Mean	79.4901	79.6339	75.4291	75.4400	51.0917	50.6068	89.2986	89.5836	57.6945	56.8371
Standard deviation	0.4659	0.2686	0.7463	0.5422	0.8348	0.4825	0.3502	0.3538	1.1421	0.5178

## Data Availability

The original contributions presented in the study are included in the article, further inquiries can be directed to the corresponding author.
